# Anamnestic humoral correlates of immunity across SARS-CoV-2 variants of concern

**DOI:** 10.1128/mbio.00902-23

**Published:** 2023-08-03

**Authors:** Ryan P. McNamara, Jenny S. Maron, Julie Boucau, Vicky Roy, Nicholas E. Webb, Harry L. Bertera, Amy K. Barczak, The Positives Study Staff, Nicholas Franko, Jennifer K. Logue, Megan Kemp, Jonathan Z. Li, Ling Zhou, Ching-Lin Hsieh, Jason S. McLellan, Mark J. Siedner, Michael S. Seaman, Jacob E. Lemieux, Helen Y. Chu, Galit Alter

**Affiliations:** 1 Ragon Institute of MGH, MIT, and Harvard, Cambridge, Massachusetts, USA; 2 Department of Medicine, Massachusetts General Hospital, Boston, Massachusetts, USA; 3 Harvard Medical School, Boston, Massachusetts, USA; 4 Department of Medicine, Brigham and Women’s Hospital, Boston, Massachusetts, USA; 5 Division of Allergy and Infectious Diseases, University of Washington, Seattle, Washington, USA; 6 Department of Molecular Biosciences, University of Texas at Austin, Austin, Texas, USA; 7 Beth Israel Deaconess Medical Center, Boston, Massachusetts, USA; 8 The Broad Institute, Cambridge, Massachusetts, USA; University of North Carolina at Chapel Hill, Chapel Hill, North Carolina, USA; University of North Carolina at Chapel Hill, Chapel Hill, North Carolina, USA

**Keywords:** vaccines, COVID-19, breakthrough, SARS-CoV-2, omicron, delta, antibodies

## Abstract

While immune correlates against SARS-CoV-2 are typically defined at peak immunogenicity following vaccination, immunologic responses that expand selectively during the anamnestic response following infection can provide mechanistic and detailed insights into the immune mechanisms of protection. Moreover, whether anamnestic correlates are conserved across variants of concern (VOC), including the Delta and more distant Omicron VOC, remains unclear. To define the anamnestic correlates of immunity, across VOCs, we deeply profiled the humoral immune response in individuals infected with sequence-confirmed Delta or Omicron VOC after completing the vaccination series. While limited acute N-terminal domain and receptor-binding domain (RBD)-specific immune expansion was observed following breakthrough infection, a significant immunodominant expansion of opsonophagocytic Spike-specific antibody responses focused largely on the conserved S2-domain of SARS-CoV-2 was observed. This S2-specific functional humoral response continued to evolve over 2–3 weeks following Delta or Omicron breakthrough, targeting multiple VOCs and common coronaviruses. Strong responses were observed on the fusion peptide (FP) region and the heptad repeat 1 (HR1) region adjacent to the RBD. Notably, the FP is highly conserved across SARS-related coronaviruses and even non-SARS-related betacoronavirus. Taken together, our results point to a critical role of highly conserved, functional S2-specific responses in the anamnestic antibody response to SARS-CoV-2 infection across VOCs. These humoral responses linked to virus clearance can guide next-generation vaccine-boosting approaches to confer broad protection against future SARS-related coronaviruses.

**IMPORTANCE:**

The Spike protein of SARS-CoV-2 is the primary target of antibody-based recognition. Selective pressures, be it the adaption to human-to-human transmission or evasion of previously acquired immunity, have spurred the emergence of variants of the virus such as the Delta and Omicron lineages. Therefore, understanding how antibody responses are expanded in breakthrough cases of previously vaccinated individuals can provide insights into key correlates of protection against current and future variants. Here, we show that vaccinated individuals who had documented COVID-19 breakthrough showed anamnestic antibody expansions targeting the conserved S2 subdomain of Spike, particularly within the fusion peptide region. These S2-directed antibodies were highly leveraged for non-neutralizing, phagocytic functions and were similarly expanded independent of the variant. We propose that through deep profiling of anamnestic antibody responses in breakthrough cases, we can identify antigen targets susceptible to novel monoclonal antibody therapy or vaccination-boosting strategies.

## INTRODUCTION

Despite the remarkable vaccine efficacy observed in phase 3 SARS-CoV-2 vaccine trials, the waning of vaccine-conferred immunity and the emergence of neutralizing antibody-resistant variants of concern (VOCs), such as the Delta (B.1.612) and Omicron (B.1.529), led to a rapid increase in transmission events globally (1 - 4). Yet, severe disease and death did not increase concomitantly suggesting that additional post-transmission blocking immune responses contribute to the control of infection and pathogen clearance once it has occurred (5). However, the precise immunologic correlates of immunity following breakthrough infections remain incompletely defined. Moreover, whether these correlates differ across VOCs that exhibit striking differences in sequence is unclear.

Neutralizing antibody responses were tightly linked to protective immunity in early mRNA vaccine phase 3 trials at a time when the dominant circulating strain was largely matched to the vaccine antigen-insert sequence (6 - 8). However, with the emergence of more neutralization-resistant VOCs, the predictive power of neutralization diminished, although not eliminated (2, 9). Despite VOC evasion of neutralization (10 - 12), both T-cells (13) and binding antibodies (14) were proposed as alternate immune mechanisms that could mediate post-treatment control and clearance of infection. Post-challenge correlates analyses in non-human primate vaccine models pointed to a rapid humoral anamnestic response, linked to the rapid expansion of antibody-secreting cells within the respiratory tract, giving rise to robust renewed pools of antibodies that may contribute to control and clearance of infection (15). Yet, whether these antibodies contribute to the attenuation of disease by the simple neutralization and blockade of further spread or via the recruitment of the antiviral activity of the local immune system, through Fc-effector functions, is unknown. Moreover, whether the specificity and functional activity of the anamnestic response that evolves following VOC infections are conserved may point to common or distinct mechanisms of attenuation of disease.

Thus, while vaccine-induced immune correlates of protection are most often focused on the identification of immunologic responses at peak immunogenicity, these immunologic markers may have limited consequence as mechanistic correlates of immunity since these responses may wane at the time of environmental exposure. Conversely, immunologic signatures following exposure may provide critical insights into the anamnestic response that is key to control and clearance of the infection. Here, we deeply profiled the humoral immune response in a cohort of individuals with a recent, documented Delta or Omicron SARS-CoV-2 infection. Systems Serology profiling revealed a rapid expansion of Fc-receptor binding and opsonophagocytic humoral immune responses across the VOC breakthrough infections with a consistent preference for expansion for the S2 subdomain of Spike, focused on the fusion peptides (FPs) and heptad repeat 1, that tracked with enhanced viral clearance. These data point to a critical role for S2-specific immunity as a key correlate of immunity across VOC breakthrough infections.

## RESULTS

### Breakthrough COVID-19 elicits spike subdomain humoral responses

The perpetual emergence of new SARS-CoV-2 variants of concern has led to repeated waves of viral breakthrough infections, even in recently vaccinated individuals (5). However, vaccine-induced immunity continues to provide protection against severe disease and death, as evidenced by the severe disease caused by both Delta and Omicron preferentially in unvaccinated populations (4, 16 - 18). Emerging data point to the potential importance of the anamnestic response as a key contributor to the resolution of infection (19). Given the association between non-neutralizing antibody effector profiles and natural resolution of severe disease (20), we performed systems serology on sera from individuals who had completed their vaccine series and had subsequent documented Delta (*n* = 37) or Omicron (*n* = 23) VOC breakthrough infection, both 1 week and 2–3 weeks post-infection (means = 6.3 ± 2.8 and 18.8 ± 2.9 days, respectively) aimed at defining the specific humoral properties associated with the resolution of infection. All individuals had received the primary two-dose series of an mRNA vaccine. Delta and Omicron breakthrough infections occurred from 5–357 days from vaccination and 0–12 days from symptom onset. Given the significant antigenic distance between Delta and Omicron within the Spike protein, we also sought to define whether anamnestic correlates were consistent across VOCs (21). We did this by deeply profiling the overall humoral landscape post-breakthrough (Fig. S1).

**Fig 1:**
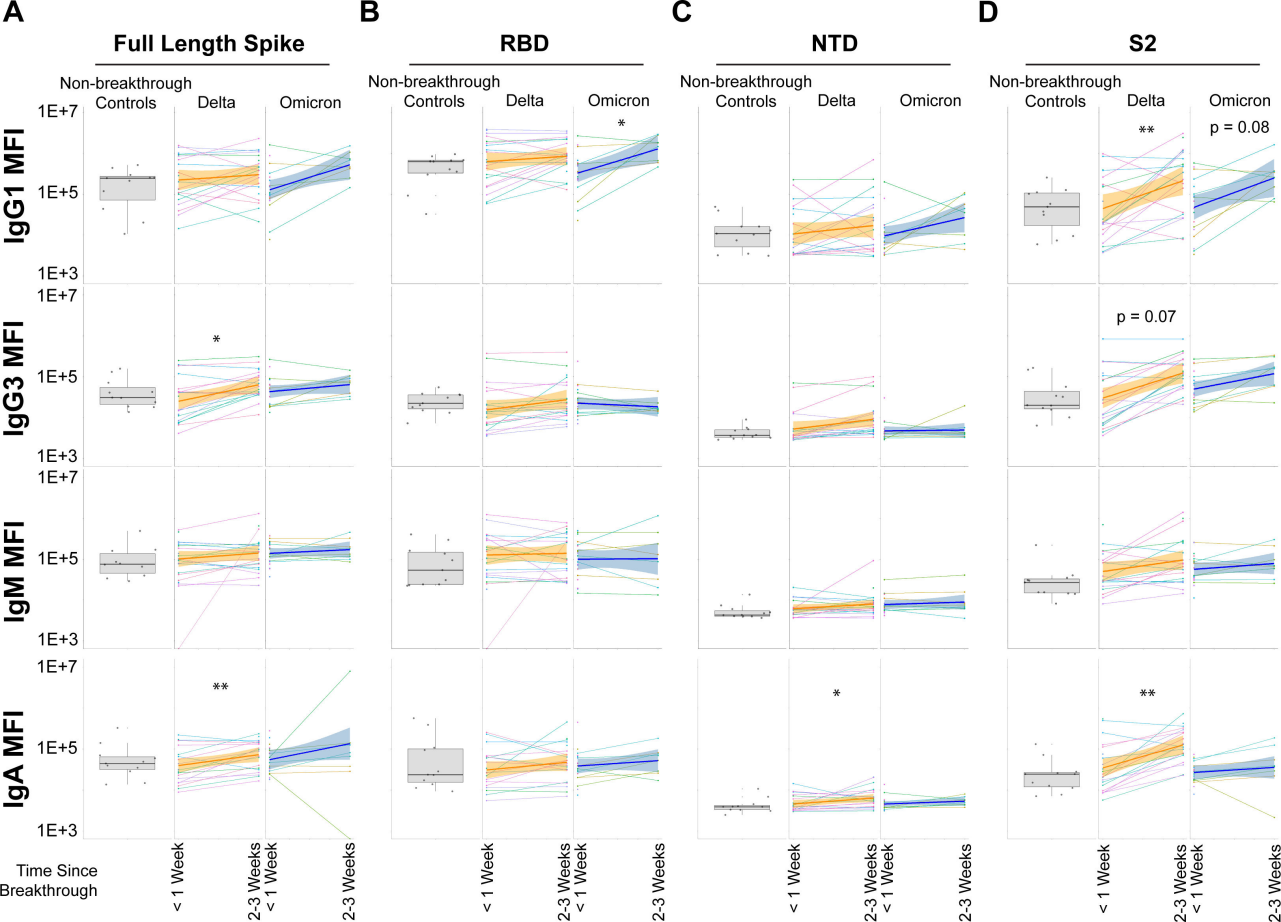
Recognition and expansion of Spike and Spike subdomains post-breakthrough infection. (**A**) Full-length, trimeric Spike was assayed for antibody recognition in non-breakthrough, vaccinated controls (gray box and whiskers, column 1), vaccinated Delta breakthrough cases at <1 week or 2–3 weeks post-infection (orange, columns 2–3), and vaccinated Omicron breakthrough cases at <1 week and or 2–3 weeks post-infection (dark blue, columns 4–5). Matched patient values are shown for breakthrough infections, along with a corresponding linear-model best-fit line (solid colored line) with 95% CI (shaded region). (**B**) Same as A but for the receptor-binding domain (RBD). (**C**) Same as A but for the N-terminal domain. (**D**) Same as A but for the S2 domain. *P < 0.05, **P < 0.01, ***P < 0.001, and ****P < 0.0001 for all panels (Mann-Whitney *U* Test; non-matched controls were not statistically grouped and are shown for reference).

The rapid expansion of Spike-specific, receptor-binding domain (RBD) and N-terminal domain (NTD)-specific immune responses has been proposed as potential acute anamnestic correlates of immunity following vaccine breakthrough infection. However, expansion of IgG1 from <1 week to 2–3 weeks for full-length Spike was limited to Delta VOC breakthrough cases and only observed for IgG3 and IgA. Non-breakthrough controls were used as reference control (gray box and whisker plots) but were not included in statistical comparisons as they were not matched (Fig. 1A). Humoral expansions for the RBD were largely muted, with the sole exception of RBD expansion for Omicron breakthrough infection for IgG1 (Fig. 1B). This is notable since the RBD is the primary subdomain targeted by neutralizing antibodies. Similarly, expansions to the NTD were quite limited, with the only observed significant increase being for Delta breakthrough infections for IgA (Fig. 1C). Expansions to S2 were more apparent, particularly for Delta breakthrough cases. IgG1 and IgA significantly expanded from <1 week to 2–3 weeks. Expansions for IgG1 recognition of S2 also trended for Omicron breakthrough infections (Fig. 1D). IgM increases were low to absent for all but one group, arguing that the majority of humoral expansions were anamnestic and not *de novo*. These data point to an unexpected and selective expansion of humoral immune responses to the highly conserved S2 domain of the Spike antigen as a key correlate of immunity following vaccine breakthrough infection.

**Fig 2:**
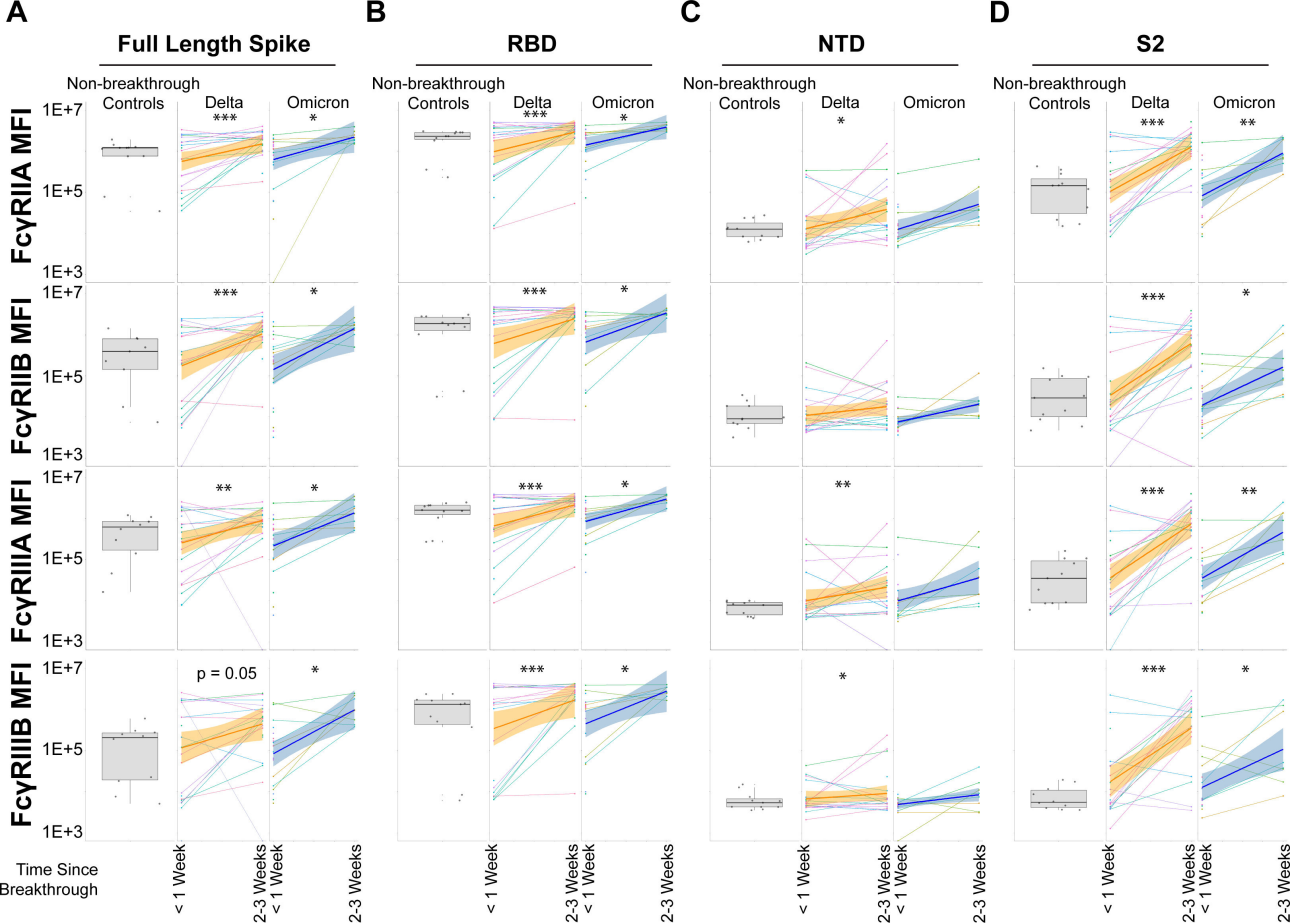
Spike and subdomain expansion of FcγR-binding antibodies post-breakthrough infection. (**A**) Full-length, trimeric Spike was assayed for FcγR-binding antibodies in non-breakthrough, vaccinated controls (gray box and whiskers, column 1), vaccinated Delta breakthrough cases at <1 week or 2–3 weeks post-infection (orange, columns 2–3), and vaccinated Omicron breakthrough cases at <1 week and 2–3 weeks post-infection (dark blue, columns 4–5). Matched patient values are shown for breakthrough infections, along with a corresponding linear model best-fit line (solid colored line) with 95% CI (shaded region). (**B**) Same as A but for Fcγ-receptor (FcγR) recognition of the RBD. (**C**) Same as A but for the NTD. (**D**) Same as A but for the S2 domain. *P < 0.05, **P < 0.01, ***P < 0.001, and ****P < 0.0001 for all panels (Mann-Whitney *U* Test; non-matched controls were not statistically grouped and are shown for reference).

Binding antibodies alone do not mediate immunologic clearance; thus, we next probed whether the anamnestic expansion of antibodies also possessed the ability to bind to Fc-receptors (FcR), the key to leveraging non-neutralizing innate immune effector functions (22, 23). All FcγR-binding antibodies profiled (FcγRIIA, FcγRIIB, FcγRIIIA, and FcγRIIIB) were significantly expanded in Omicron VOC breakthrough infections for the full-length, trimeric spike similar to Delta breakthrough infections; the sole exception was FcγRIIIB for Delta VOC breakthrough cases, which trended strongly toward expansion but did not reach statistical significance (Fig. 2A). Expansions toward the RBD were consistently significant across VOCs and across FcγRs (Fig. 2B). Notably, NTD expansions of FcγR-binding antibodies were absent in Omicron breakthrough infections and inconsistent for Delta breakthrough infections (Fig. 2C). This is in agreement with binding antibodies. In contrast, FcγR-binding antibodies to S2 were strongly expanded for both Delta and Omicron breakthrough infections (Fig. 2D).

The temporal-based increase in Fab and Fc-binding antibodies was exclusive for SARS-CoV-2 antigens as no increases were detected for control antigens (Fig. S2), demonstrating the specificity of our approach.

### Recognition of S2 is expanded in breakthrough cases

**Fig 3:**
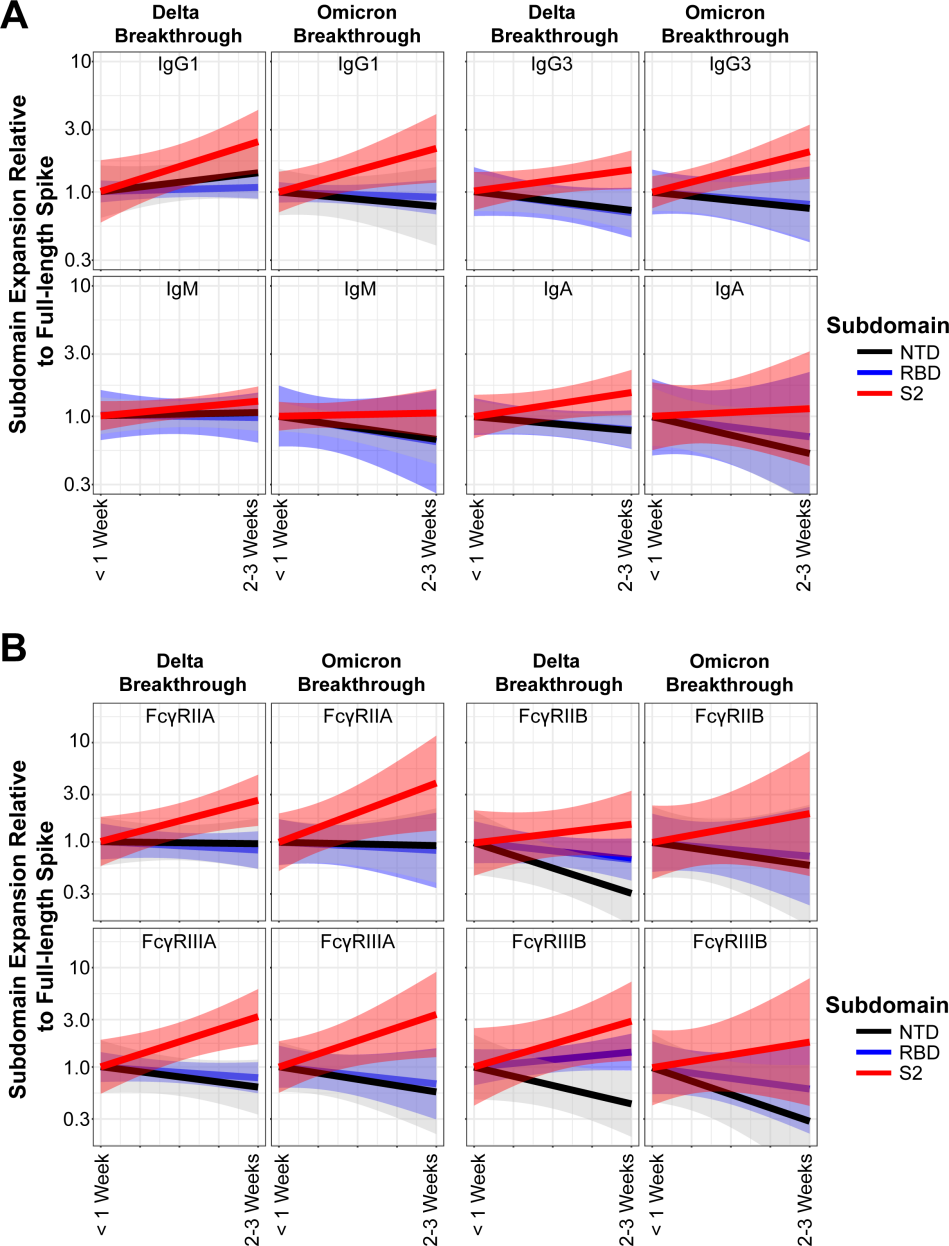
The S2 subdomain drives the majority of humoral expansions post-breakthrough infection. (**A**) Fold expansions of subdomains of Spike were plotted relative to expansions of full-length trimeric Spike for IgG1, IgG3, IgA, and IgM. A value of 1 indicates the same expansion rate of a subdomain as full-length Spike, a value >1 indicates an accelerated expansion relative to full-length Spike, and a value <1 indicates an expansion reduced compared to full-length Spike. Shown are the best-fit linear models of matched patients with 95% CI in shaded regions. (**B**) Same as A but for FcγR-binding antibodies toward the Spike subdomains.

To directly compare the extent of the anamnestic expansion across Spike domains in all the breakthrough cases, we examined the fold increase in IgG1 levels specific to subdomains of Spike. We standardized each patient’s response to full-length Spike expansion to identify subdomains that were driving the overall expansion. We set the *y*-intercept to 1 for <1 week post-infection for all subdomains and then assayed for fold expansion between that day and the 2- to 3-week post-infection timepoint (see Materials and Methods). IgG1 expansion for both Delta and Omicron breakthrough infections skewed toward S2 as driving the total change (red line), whereas expansions to the RBD and NTD tracked similarly to total Spike expansion. Both IgG3 and IgA showed similar profiles in subdomain-specific expansion for both VOCs. IgM expansions for S2 were flat when standardized to full-length Spike for both VOCs, again arguing against *de novo* humoral responses (Fig. 3A). The same approach was taken for visualizing subdomain-specific FcγR-binding antibodies for both VOCs. Similar to binding antibodies, FcγR-binding antibody expansions skewed toward S2 for both VOCs for all FcγRs assayed. In agreement with our previous findings, expansions of NTD were either linearly related to total Spike expansion or lagged behind. A positive RBD expansion for FcγRIIIB for Delta breakthrough cases was observed, but that was the only notable RBD expansion for either VOC (Fig. 3B).

These results indicate that anamnestic responses, and not *de novo*, drive humoral profiles post-breakthrough with VOCs. Moreover, anamnestic responses are disproportionately driven by the S2 domain of Spike.

### S2 is the subdomain that drives humoral expansion post-breakthrough infection

**Fig 4:**
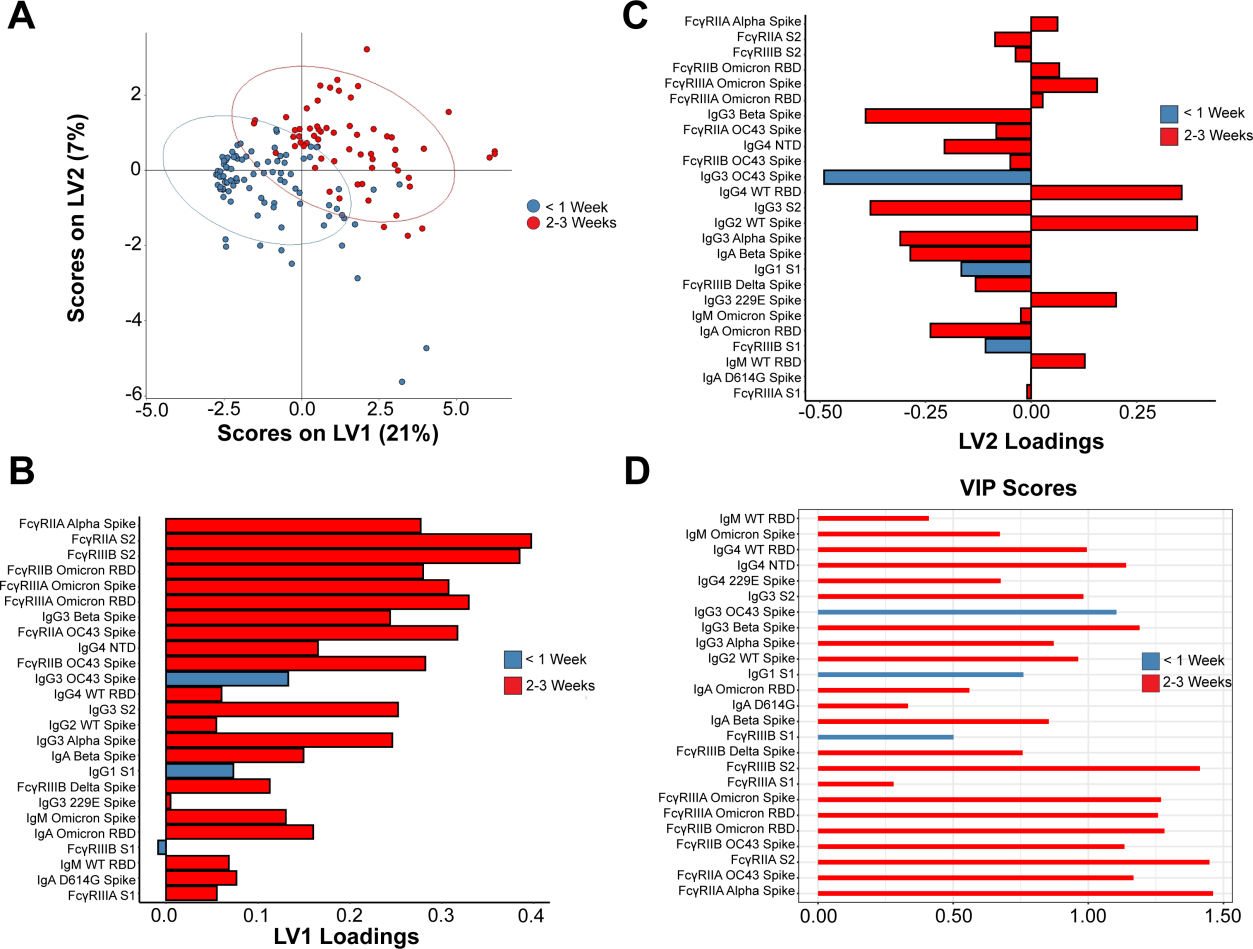
Expansion of S2 recognition is a marker for breakthrough COVID-19. (**A**) Partial least squares determinant analysis clustering of humoral profiles of vaccinated individuals who experienced COVID-19 breakthrough from Delta or Omicron VOC at <1 week post-infection (blue) and 2–3 weeks post-infection (red) (**B**) Ranked sum of identified features in the PLS-DA on the LV1 axis, which is the major axis of separation; bars in blue correspond to selected features in breakthrough cases <1 week and red for features at 2–3 weeks post-infection. (**C**) Same as B but for the LV2 (minor) axis of separation. (**D**) Variable importance of protection scores of the PLS-DA.

Given that univariate comparisons of anamnestic responses between Delta and Omicron breakthrough cases were highly similar, we next sought to define a minimal multivariate signature of breakthrough infection for both VOCs. We, thus, performed a partial least squares discriminant analysis (PLS-DA) on antibody responses of breakthrough cases at <1 week post-infection (blue circles) and 2–3 weeks post-infection (red circles). Using a least absolute shrinkage and selection operator-based thresholding, time-based clustering was evident in the COVID-19 breakthrough infections, mostly along latent variable 1 (LV1) (Fig. 4A). Minimal features responsible for group separation were identified, along with their corresponding LV1 (Fig. 4B) and LV2 scores (Fig. 4C), and model validation (24, 25) was confirmed against randomly selected features and permutated labels (Fig. S3). Multiple FcγR-binding antibodies toward subdomains and full-length Spike proteins of VOCs were enriched in the 2- to 3-week post-breakthrough group. Binding antibodies exhibited a similar trend toward enrichment within the 2- to 3-week post-breakthrough group. Plotting variable importance of protection (VIP) scores of the multivariate clustering revealed a strong preference for FcγR-binding antibodies, with full-length Spike as well as RBD and S2 subdomain targeting antibodies exerting the highest scores for the 2- to 3-week post-breakthrough group. Of the NTD-specific antibody responses, only NTD-specific IgG4 responses were preferentially enriched among the 2- to 3-week post-breakthrough group. Specifically, a coordinated expansion of neutrophil-specific FcγRIIIB and opsonophagocytic FcγRIIA binding antibodies was selectively expanded in breakthrough infection profiles. Additionally, S2-specific IgG3 responses expanded preferentially in the 2- to 3-week grouping (Fig. 4D).

A near-identical pattern of S2 expansion in Fab and FcγR-binding antibodies was observed in a second, independent cohort across Delta and Omicron breakthrough infections after 30 days compared to reference controls (Fig. S4). These data collectively highlight the broader selective expansion of functional FcγR-binding responses, largely focused on S2, across both Delta and Omicron breakthrough infections.

### Breakthrough infection drives a functional S2-specific humoral immune expansion

**Fig 5:**
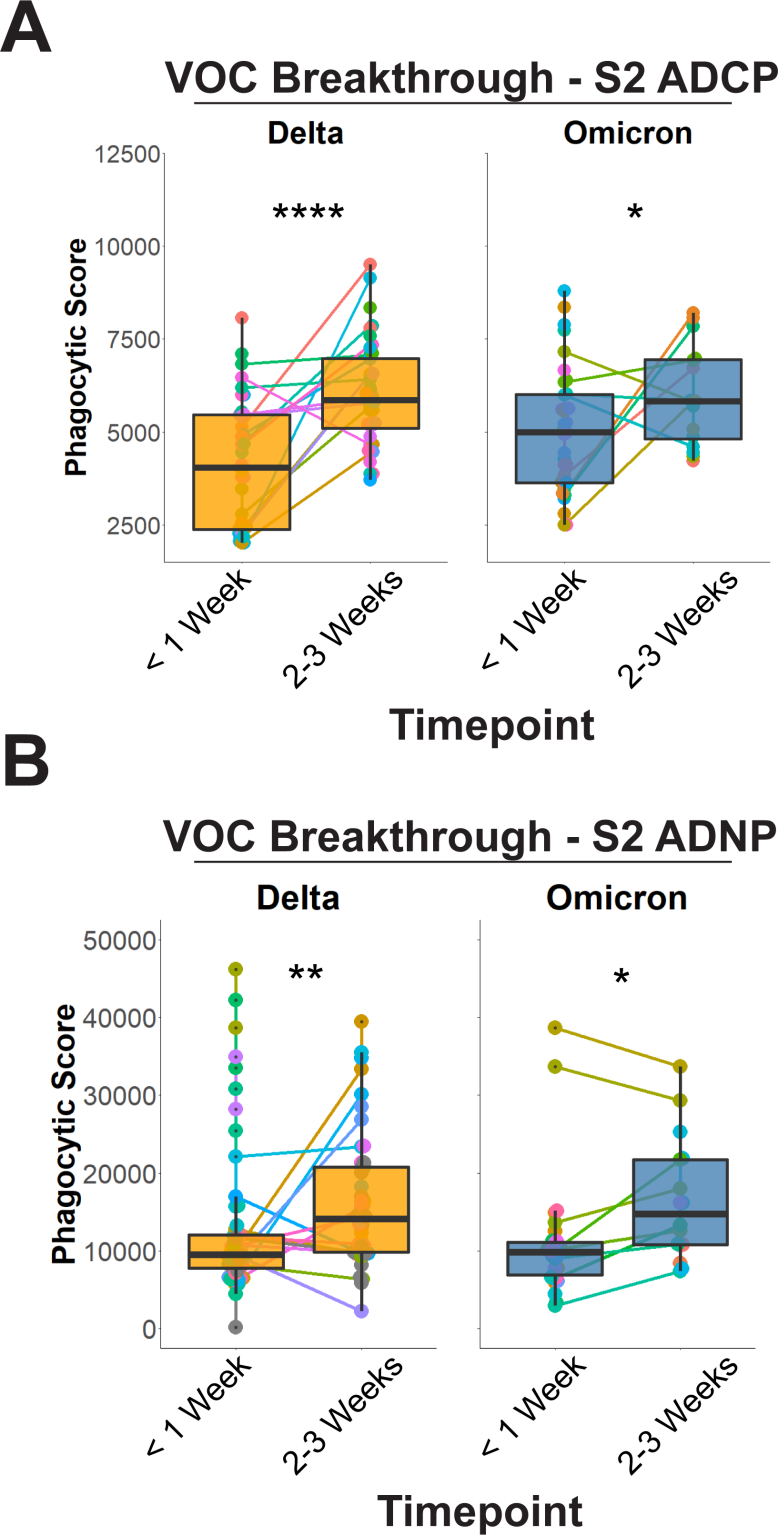
Expansion of S2 recognition is functionally linked to antibody-mediated opsonophagocytic activity. (**A**) Antibody-mediated cellular phagocytosis by monocytes was quantified using sera from (left, orange) Delta breakthrough cases at <1 week or 2–3 weeks post-infection and (right, blue) Omicron breakthroughs at <1 week or 2–3 weeks post-infection for the S2 domain alone (right). (**B**) Same as A,but for antibody-dependent neutrophil phagocytosis (ADNP) of S2. *P < 0.05, **P < 0.01, ***P < 0.001, and ****P < 0.0001 for all panels (Mann-Whitney *U* Test).

Whether the expansion of S2-specific immunity is simply a biomarker of exposure to the virus or a mechanistic correlate of immunity is unclear. However, binding alone is insufficient to drive antiviral control or clearance; thus, we next aimed to determine the functional capacity of the breakthrough infection anamnestic response. Critically, among the antibody effector functions, we previously observed a preferential expansion of Spike-specific opsonophagocytic activity in natural survivors of severe disease (23) and, thus, we profiled S2-specific antibody-dependent monocyte and neutrophil phagocytic profile. A significant increase in S2-specific antibody-dependent cellular monocyte phagocytic (ADCP) activity was observed across the Delta and Omicron breakthrough cases (Fig. 5A).

Antibody-dependent neutrophil phagocytosis (ADNP) has been linked to the natural resolution of infection, convalescent plasma therapeutic activity, and vaccine-mediated immunity (20, 26). ADNP is tightly linked to FcγRIIIB responses, and given that S2-dependent ADNP was an identified feature distinguishing responses over time, we sought to confirm this at the univariate and functional levels. Similar to ADCP, S2-dependent ADNP significantly expanded 2–3 weeks post-breakthrough infection for both VOC (Fig. 5B).

Expansions of neutralizing antibody levels were also investigated in the context of S2 expansions. Neutralization expansion of Spike post-breakthrough infection with Delta increased but was correlated exclusively with the RBD domain (27). No correlations were observed between neutralizing antibody titer and NTD- or S2-directed antibodies. Interestingly, no correlations were observed between Omicron neutralizing antibody titer and Spike, RBD, NTD, or S2-directed antibodies (Fig. S5). These results further support that S2 expansions in breakthrough cases are linked to non-neutralizing functions leveraged by the Fc-domain. Moreover, given that the rate of S2-directed antibody expansions outpaced other subdomain-directed antibodies further highlights that anamnestic responses are primarily leveraged through non-neutralizing humoral responses.

### Selective expansion of fusion peptide and heptad repeat 1-specific immunity

To define whether S2-specific responses targeted particular regions of S2, we finally mapped the expanding breakthrough infection response across peptides spanning the S2 domain of the Spike antigen by quantifying relative IgG1 binding. We were specifically interested in the fusion peptides and the heptad repeat regions given their conservation among sarbecoviruses (28 - 30), previously described antigenic potential including potentially neutralization targets (31 - 34), and their differential exposure status based on pre- and post-fusion conformational rearrangements (35, 36).

**Fig 6:**
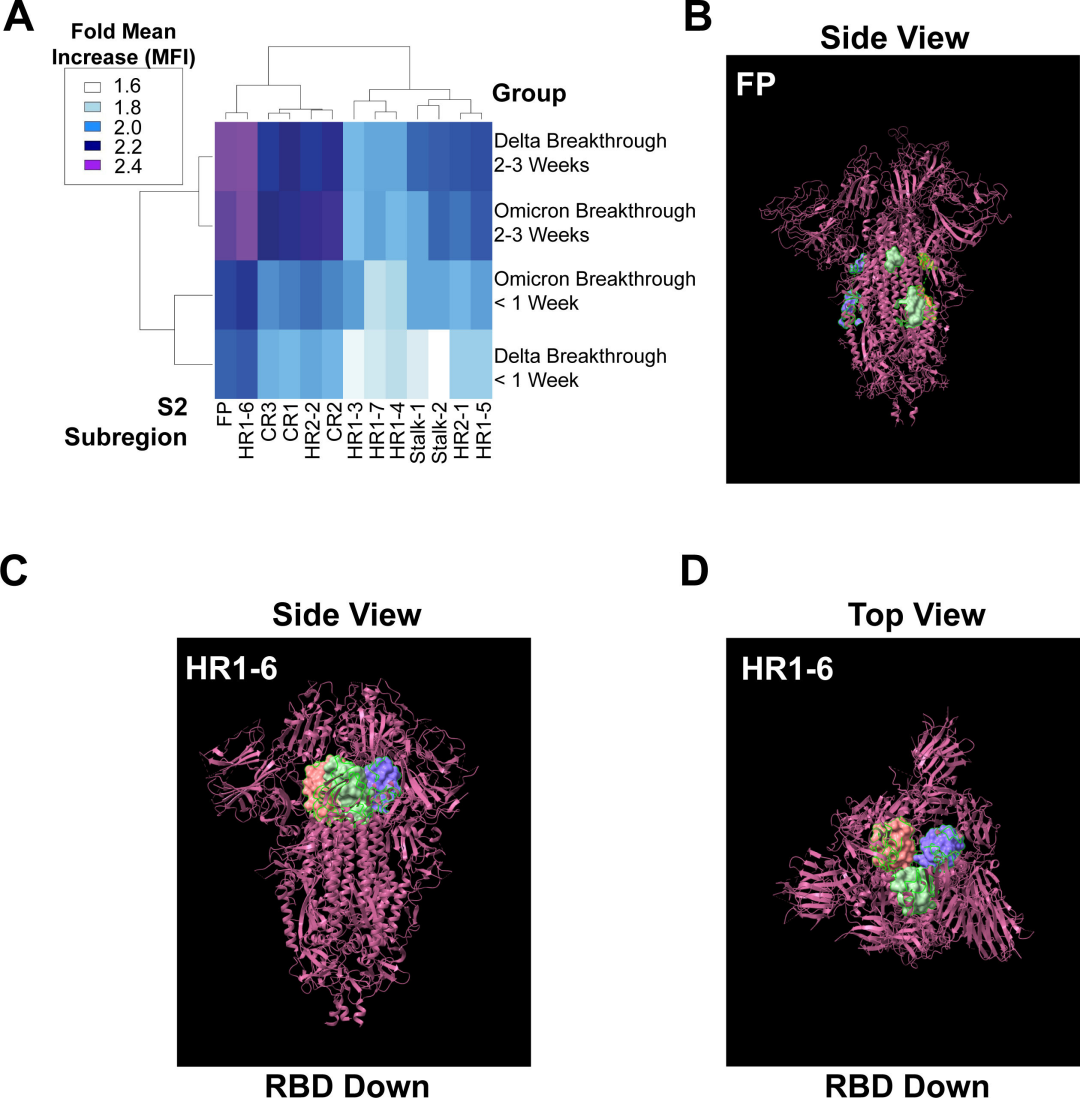
Immunodominant expansion of S2 is focused on the fusion peptide and heptad repeat region 1 for VOC breakthrough infections. (**A**) Clustered heatmap of IgG1 binding to peptides spanning subregions of S2 post-breakthrough Delta and Omicron infections during the <1-week and 2- to 3-week post-infection responses; heatmap legend is shown on the left. Binding was standardized to non-infected reference controls. (**B**) The fusion peptide region was highlighted and displayed on the 3-D Chimera ×1.4 software on the SARS-CoV-2 D614G Spike Trimer (magenta). (**C**) The HR1-6 region is highlighted as a part of the SARS-CoV-2 D614G Spike Trimer (magenta) when viewed from the side in the RBD-down conformation. (**D**) Same as C but from the perspective of the top of the protein, furthest away from the surface of the viral particle.

A blinded clustering revealed a highly focused and VOC-independent response to the FP and a subregion within HR1 (Fig. 6A) across both Delta and Omicron breakthrough cases. It was notable that these responses were higher even at <1 week post-breakthrough infection and continued to expand over time. At the structural level, the FP region is partially exposed on the S2 when Spike is in its folded, trimeric form (37, 38) (Fig. 6B). Other reports have identified FP-targeting antibodies (33, 39). Interestingly, the region in HR1 that exhibited disproportionate IgG1 binding was directly adjacent to the RBD and sterically hindered in the RBD-down conformation (Fig. 6C and D). While seemingly counter-intuitive to antibody targeting given the folding of the Spike trimer, other HR1-binding antibodies have been reported (28, 31, 34, 40).

Lastly, we probed whether responses to particular subregions within S2 were correlated with virus clearance. Comparison of SARS-CoV-2 RNA decay slopes with S2-peptide-specific IgG expansions for the first 3 weeks of breakthrough infection pointed to a highly significant inverse correlation between the slope of the evolution of FP-specific responses and viral clearance but only at the 2- to 3-week post-breakthrough timepoint (Fig. S6). Additionally, other S2-peptide-specific responses were also significantly associated with viral clearance after 2–3 weeks including regions within HR1, pointing to a potentially critical role of multiple S2-specific responses in the elimination of viral replication. Collectively, these data point to a common immunodominant S2 anamnestic response to FP, and the heptad repeats that may form the basis of a rapid functional humoral response required to capture, contain, and eliminate VOCs until T cells are able to traffic, expand, and eliminate remaining virus to ultimately clear the disease.

## DISCUSSION

Correlates of immunity represent immunological biomarkers that are statistically enriched in individuals that exhibit protective immunity in vaccine trials (20, 29, 41 - 44). Correlates may be mechanistically involved in protective immunity but can also represent surrogates of other immunological mechanisms key to anti-pathogen control. Most correlates of immunity are defined at the time of peak immunogenicity, aimed at defining immune responses able to predict clinical outcomes. However, within most trials, infections occur over prolonged periods of time, and vaccine-induced immune responses may have waned from peak immunogenicity. Thus, the immunological markers associated with protection over time may differ from those observed at peak immunogenicity. However, the collection of samples prior to infection over time in large Phase 3 trials is cost-prohibitive. Instead, the analysis of responses that selectively expand soon after infection can offer additional insights into the mechanism(s) by which vaccine-induced immunity reacts to control a challenge.

Phase 3 peak immunogenicity correlates analyses pointed to a strong association between vaccine-induced Spike-specific neutralizing and binding antibody titers with protection across the mRNA platforms (6, 8). Here, we profiled the post-infection immunological profiles that evolved in breakthrough infections, with a unique interest in defining whether the kinetics of breakthrough correlates of immunity were consistent across variants of concern. While an expansion of full-length Spike IgG responses was observed in both Delta and Omicron breakthrough infections, limited expansion was observed in NTD-specific titers across both groups. Instead, the majority of the Spike-specific expansion was related to a unique anamnestic expansion of early S2 FP and HR1-specific IgM antibodies able to leverage monocyte phagocytosis, followed by a more mature S2 FP- and HR1-specific IgG FcR-binding neutrophil recruiting response observed in both Delta and Omicron breakthrough infections. Thus, despite the immunodominant vaccine-induced response to the RBD, these data point to a critical and unexpected role of S2-specific functional humoral immunity as critical anamnestic correlates of immunity across VOCs.

Early studies of immune correlates of the natural resolution of COVID-19 pointed to a selective expansion of S2-specific functional humoral immunity in survivors of natural severe COVID-19 infection. S2-specific antibodies were noted in survivors of severe COVID-19 from the time of intensive care unique admission (23, 44). Moreover, expanded S2-specific humoral immune responses were also noted in children (29), individuals that developed milder forms of COVID-19, as well as in individuals with asymptomatic infections (44). Interestingly, these natural S2-specific humoral immune responses may have emerged from pre-existing common-coronavirus-specific humoral immune responses, that also expanded, marked by preferential FcR binding, among individuals with asymptomatic infection. Common β-coronaviruses are largely conserved in their S2 domains. S2-specific monoclonals exhibit cross-coronavirus reactivity and *in vivo* protection in an Fc-dependent manner (28), arguing that these less potent neutralizing antibodies target a highly conserved region of the SARS-CoV-2 Spike may depend on non-neutralizing mechanisms of action. Given the robust association between pre-existing common-coronavirus immunity and attenuated COVID-19 (29), these data point to a critical role for cross-reactive S2-specific humoral immunity in both infection and vaccine-induced immune responses.

The S2 domain includes the fusion machinery required for viral entry (45), requiring precise packaging and movement upon Spike binding to the host angiotensin-2 (ACE2) receptor. Thus, unlike other regions of the Spike antigen, S2 is less mutable and has shown conservation across distinct VOCs, with only ~2 and 12 mutations in Delta and Omicron, respectively (depending on sublineage), representing the most highly conserved domain of the SARS-CoV-2 Spike antigen. However, the S2-directed response is less dominant following mRNA vaccination, likely related to the two proline stabilization introduced in the protein to stabilize the antigen during vaccination (46, 47). This stabilization holds S1 and S2 in a pre-fusion state, likely required to drive robust immunity to the primary target of neutralizing antibodies, the RBD. However, this stabilization also may make S2 less accessible to the immune response. Infection with the virus generates copious amounts of Spike in its inherently destabilized form. This allows immune surveillance networks to sample both S1 and S2 domains in pre- and post-fusion forms, triggering an anamnestic response. The differences in the anamnestic immunodominance of S1 and S2 may relate to the fact that S1 is presented largely as a soluble protein, whereas S2 may be presented in a particulate form due to the C-terminal transmembrane anchoring domain of the protein. Conversely, S2-responses may gain a competitive advantage due to the high degree of conservation across VOCs, able to recruit pre-existing B cells, whereas previously programmed RBD- and NTD-specific B cells may struggle to bind to the incoming VOC due to significant antigenic variation (1, 2, 4). However, despite the enhanced sequence conservation between the vaccine strain and Delta compared to Omicron, S2-specific immunity expanded in both Delta and Omicron breakthrough infections, suggesting that S2-specific anamnestic correlates may be key to protection across VOCs.

Our approach demonstrated that subregions within S2 displayed different anamnestic responses, with the FP and a subregion of HR1 adjacent to the RBD having the highest recall responses. We cannot discount, however, that other subregions within S2 exist that drive anamnestic responses as our peptide scanning array did not cover the entirety of S2. Rather, we profiled subdomains within S2 that have previously been shown to have antibody responses, including some neutralizing functions (32, 34, 36). Moreover, our approach cannot rule out the contribution of non-linear S2 epitopes, such as those formed in the context of the native, trimeric state of Spike, as contributors to anamnestic responses. Future work using natively folded, trimeric state S2 in various conformations would be highly informative.

Expansion of S2-specific immunity following breakthrough infection was independent of the vaccination platform. Therefore, while humoral profiles can be distinguished by the type of vaccination received, it is important to note that these recall responses were conserved. Thus, strategies to boost functional humoral immunity to expanded regions within Spike may represent a promising future strategy to maintain long-term protection against severe disease and death against current and future VOCs regardless of primary vaccination series. Whether these S2-specific responses can work in concert with T cells that also target conserved regions of the SARS-CoV-2 Spike remains unclear (13); however, these responses point to important, unexpected targets of the immune response that may be key to the durable protection against disease severity. Moreover, these regions that show higher conservation between VOCs, and β-coronaviruses in general, that are expanded post-infection could be viewed as rational vaccination booster targets.

## MATERIALS AND METHODS

### Contact for reagent and resource sharing

Further information and requests for resources should be directed to the data point of contact, Ryan McNamara (rpmcnamara@mgh.harvard.edu).

### Experimental model and subject details

#### Study approval

Approval for the study of breakthrough COVID-19 at Mass General Brigham was approved under protocol 2021P000812 by the Mass General Brigham IRB. For this cohort, ambulatory individuals in the Mass General Brigham healthcare system with a positive SARS-CoV-2 test were recruited and provided nasal swabs for self-collection three times weekly (Table S1). Sequencing from anterior nasal swabs was performed for VOC identification. Blood was drawn at enrollment and approximately 2 weeks later for acute and convalescent immune response measurement. All study participants provided informed consent. The use of healthy donor blood for cellular functional assays is approved under protocol 2021P002628 by the Mass General Institutional IRB.

The Hospitalized or Ambulatory Adults with Respiratory Viral Infections study was approved by the University of Washington Human Subjects Division Institutional Review Board (STUDY00000959).

### Method details

#### Antigens

All antigens and peptides used in this study are listed in Table S1 and Table S2. The protein antigens were received in lyophilized powder form and resuspended in water to a final concentration of 0.5 mg/mL. Peptides were received in solution and, if necessary, were buffer exchanged using Zeba-Spin columns (ThermoFisher, USA).

#### Immunoglobulin isotype and Fc receptor binding

Sera was collected from participants at two timepoints post-COVID-19 diagnosis, with the first timepoint being 6.3 ± 2.8 days post-observed start date and 18.8 ± 2.9 days post-observed start date. The two groups were clustered together for analyses and classified as <1 week and 2–3 weeks.

Systems serology for antigen-specific recognition was done using custom multiplex magnetic Luminex beads (Luminex Corp, Austin, TX, USA) as previously described (22). Serum samples were heat inactivated at 56°C for 60 min and then centrifuged at 13,000 × *g* for 5 min. Pelleted material was discarded, and clarified sera were placed into a 96-well plate. Antigens and peptides were coupled to beads through carbodiimide-NHS ester-coupling chemistry. The antigen- or peptide-coupled beads were incubated with diluted heat-inactivated serum (1:100 for IgG2, IgG3, IgG4, IgM, and IgA1; 1:250 for IgG1; and 1:750 for Fcγ receptor binding; all dilutions were in 1X PBS) overnight at 4°C in 384 well plates (Greiner Bio-One, Frickenhausen, Germany). Secondary antibodies were PE conjugated and incubated with samples at room temperature for 1 h at a 1:100 dilution in sterile-filtered Assay Buffer (1× PBS, pH 7.4, 0.1% BSA, 0.05% Tween 20). For Fcγ-receptors, PE-streptavidin (Agilent Technologies, CA, USA) at a 1:1000 dilution.

For flow cytometry analysis, the IQue Screener PLUS cytometer (IntelliCyt) was used using customized gating for each bead region. Fluorescence in the BL2 channel was quantified and exported into .csv files and subsequently analyzed (see below).

#### Subdomain expansions quantitation

To examine how subdomains were contributing to the total expansion of Spike, we divided the mean fluorescence intensity (MFI) of each patient’s subdomain response by their total Spike response for all antibody isotypes, subclasses, and FcγR-binding antibodies. We then plotted the expansions using a moving average from the <1-week timepoint to the 2- to 3-week timepoint. Shaded regions indicate the 95% confidence interval. Only patients who had values for both timepoints were included. Y-intercepts were set to 1 at *T* = <1 week. In this model, a value >1 indicates that the subdomain is expanding at a rate greater than total Spike expansion, a value of 1 indicates a rate the same as total Spike expansion, and a value <1 indicates a rate lower than the total Spike expansion.

#### Viral loads quantitation and viral sequencing

SARS-CoV-2 RNA was extracted and quantified as previously described (21, 48). VOC identification through sequencing was done using a previously validated method (27). Peak viral loads for each patient were quantified, and the mean peak viral loads between VOCs were calculated.

#### Evaluation of antibody-mediated functions

Antibody-dependent cellular phagocytosis by monocytes and neutrophil phagocytosis were quantified using a validated, flow cytometry-based bead phagocytic assay (26). Fluorescently labeled microspheres were coupled to antigens through biotinylation and conjugation to neutravidin beads. Diluted and heat-inactivated serum samples were incubated with antigen-coupled neutravidin beads to create a pre-immune complex. The solution was then incubated with THP-1 monocytes (ATCC, Manassas, VA, USA) or primary-derived neutrophils (22). For ADNP, cells were stained with anti-CD66b Pacific blue antibody to calculate the percentage of CD66b + neutrophils. Cells were fixed with 4% paraformaldehyde. Microsphere uptake was quantified by the percentage of microsphere-positive cells × MFI of microsphere-positive cells.

### Quantification and statistical analysis

Data visualizations and analysis were done using R Studio V 1.4.1103 or GraphPad Prism. Box and whisker plots were generated using ggplot showing the mean and standard deviation for each group as factors. An initial ANOVA was performed to identify significant groupings. A Mann-Whitney *U* test was used to determine statistical grouping, followed by a multiple tests correction when necessary. For all analyses, * stands for *P* < 0.05, ** stands for *P* < 0.01, *** stands for *P* < 0.001, and **** stands for *P* < 0.0001. All codes and scripts are available upon request, and no original code was created for this manuscript.

## Data Availability

The analysis pipeline is publicly available on the systemsseRology on GitHub (GitHub - LoosC/systemsseRology: Machine learning tools for the analysis of systems serology data). Data generated for this study can be found on the RagonSystemSerology homepage on GitHub (project identifier mBio20230609).

## SUPPLEMENTAL MATERIAL

Figure S1 mbio.00902-23-s0001.pdf Antibody binding array of SARS-CoV-2 and control antigens.

Figure S2 mbio.00902-23-s0002.pdf Humoral expansions are specific for SARS-CoV-2.

Figure S3 mbio.00902-23-s0003.pdf Validation of PLS-DA generated model of distinguishing humoral features at <1 week and 2-3 weeks post-infection.

Figure S4 mbio.00902-23-s0004.pdf S2-expansion is observed in independent cohorts.

Figure S5 mbio.00902-23-s0005.pdf Correlations between neutralization and Spike domains are focused on the RBD of D614G and Delta, but not S2, post-breakthrough.

Figure S6 mbio.00902-23-s0006.pdf Humoral S2 subregion expansions are inversely correlated with viral loads.

Table S1 mbio.00902-23-s0007.docx Cohort analysis by VOC breakthrough and vaccine type.

Table S2 mbio.00902-23-s0008.docx Peptide names and sequences used for this study.

ASM does not own the copyrights to Supplemental Material that may be linked to, or accessed through, an article. The authors have granted ASM a non-exclusive, world-wide license to publish the Supplemental Material files. Please contact the corresponding author directly for reuse.
