# Supplementary material for: Anamnestic humoral correlates of immunity across SARS-CoV-2 variants of concern
Source: mBio. 2023 Aug 3;14(4):e00902-23. doi: 10.1128/mbio.00902-23 (PMC10470538; doi:10.1128/mbio.00902-23)
Supplement: Figure S1 — Antibody binding array of SARS-CoV-2 and control antigens. [file mbio.00902-23-s0001.pdf]

## Figure S1

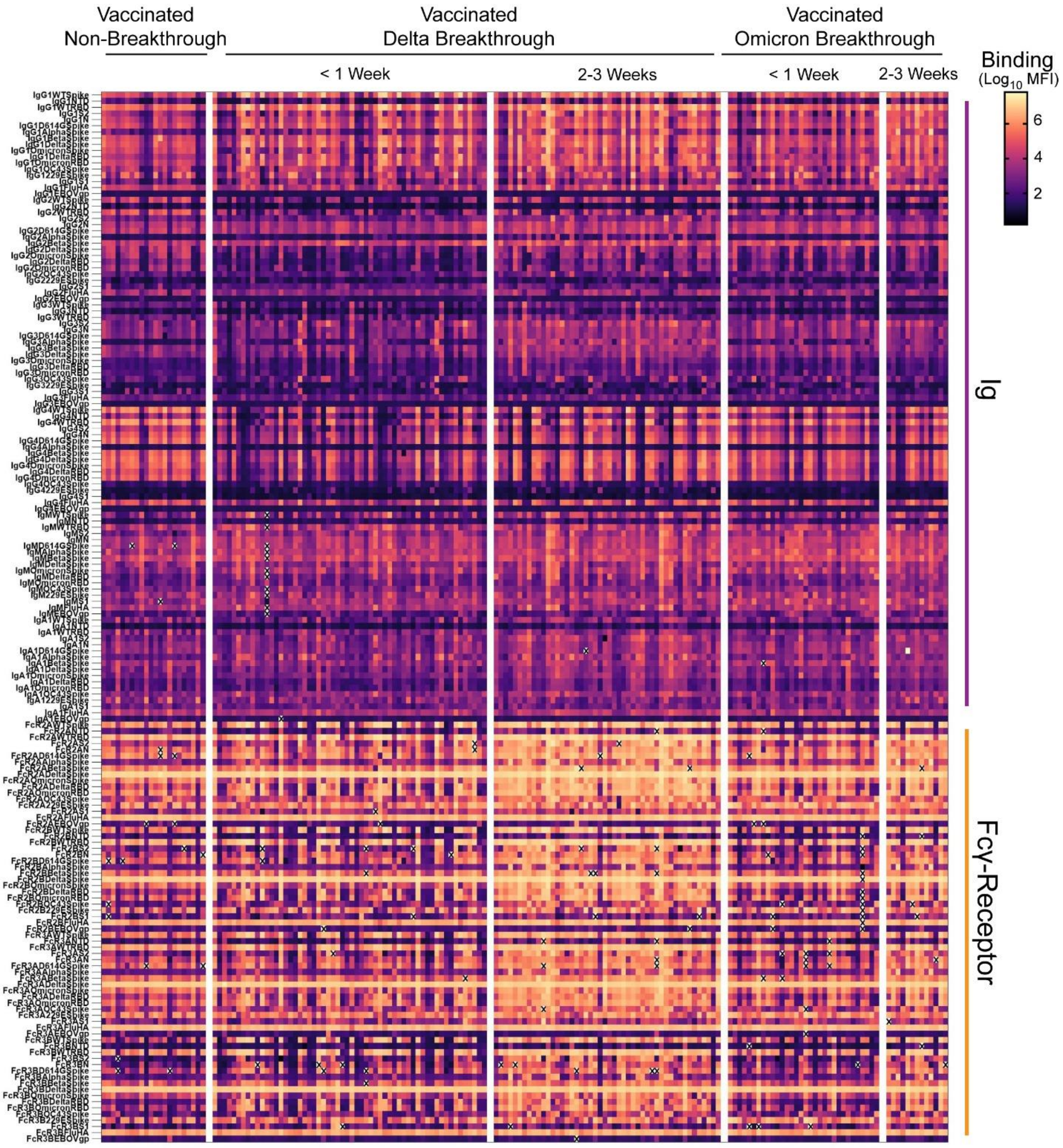


**Supplementary Figure 1. Antibody binding array of SARS-CoV-2 and control antigens.** Sera taken from non-infected, vaccinated individuals (left) and breakthrough Delta and Omicron VOCs infections were assayed for binding to various SARS-CoV-2 and control antigens. Breakthrough cases had two timepoints collected: < 1 Week post-confirmed breakthrough and 2-3 Weeks post-infection. Both Ig and FcγR-binding antibodies were quantified for antigen binding. The heatmap legend for overall binding is shown on the right.
