# Supplementary material for: Anamnestic humoral correlates of immunity across SARS-CoV-2 variants of concern
Source: mBio. 2023 Aug 3;14(4):e00902-23. doi: 10.1128/mbio.00902-23 (PMC10470538; doi:10.1128/mbio.00902-23)
Supplement: Figure S2 — Humoral expansions are specific for SARS-CoV-2. [file mbio.00902-23-s0002.pdf]

Figure S2

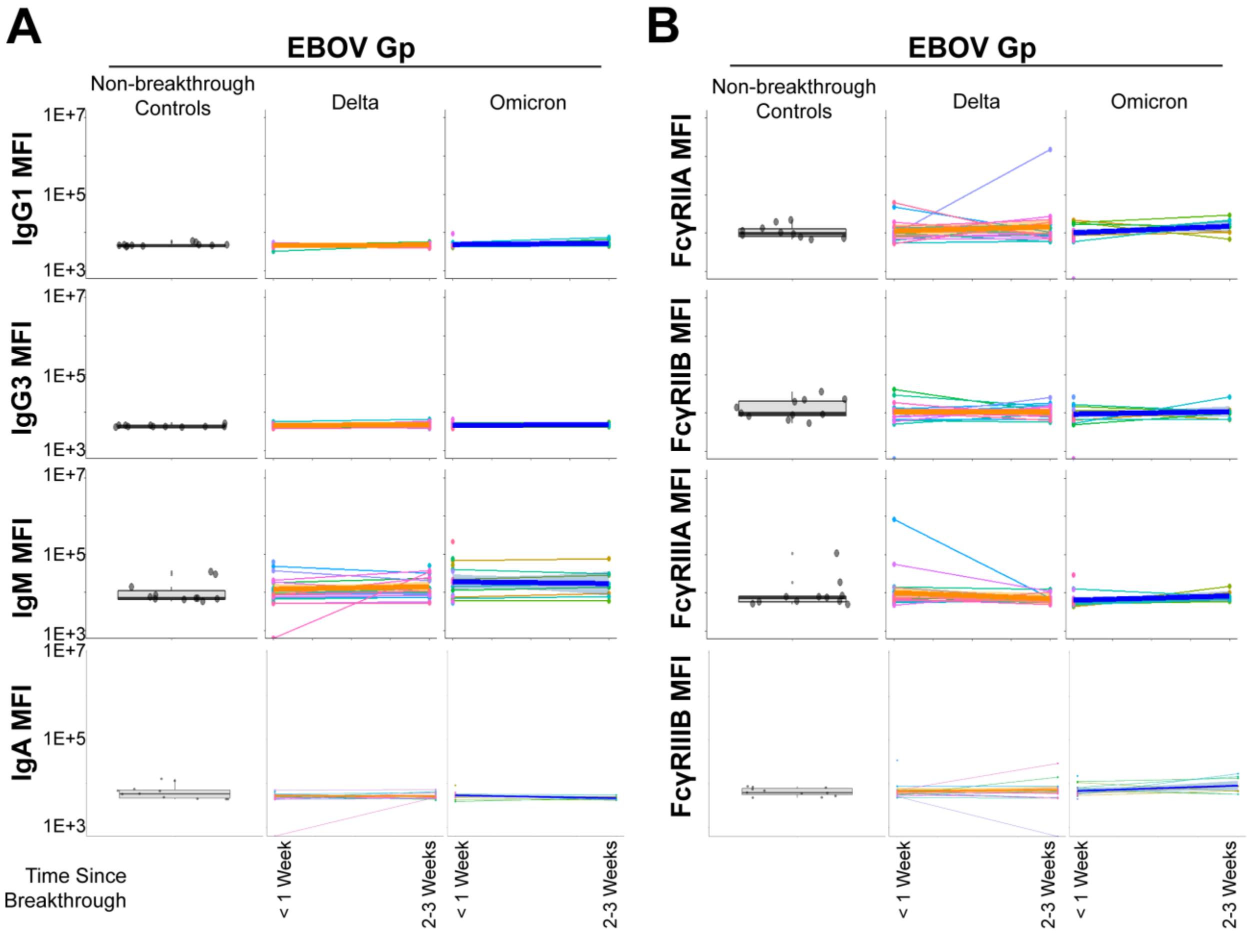

Supplementary Figure 2. Humoral expansions are specific for SARS-CoV-2. (A) Ebola virus glycoprotein (EBOV Gp) was used as an antigen-specificity control for our binding arrays. Shown are relative binding for IgG1, IgG3, IgM, and IgA for non-breakthrough controls (gray box and whisker, column 1) and Delta breakthrough cases at < 1 week and 2-3 Weeks post-infection (orange, columns 2-3) and Omicron breakthrough cases (blue, columns 4-5) at < 1 week and 2-3 Weeks post-infection. Matched patient values are shown for breakthrough infections, along with a corresponding linear-model best-fit line (solid colored line) with 95% CI (shaded region). (B) Same as A, but for FcγR-binding antibodies towards EBOV Gp.
