# Supplementary material for: Anamnestic humoral correlates of immunity across SARS-CoV-2 variants of concern
Source: mBio. 2023 Aug 3;14(4):e00902-23. doi: 10.1128/mbio.00902-23 (PMC10470538; doi:10.1128/mbio.00902-23)
Supplement: Figure S3 — Validation of PLS-DA generated model of distinguishing humoral features at <1 week and 2-3 weeks post-infection. [file mbio.00902-23-s0003.pdf]

Figure S3

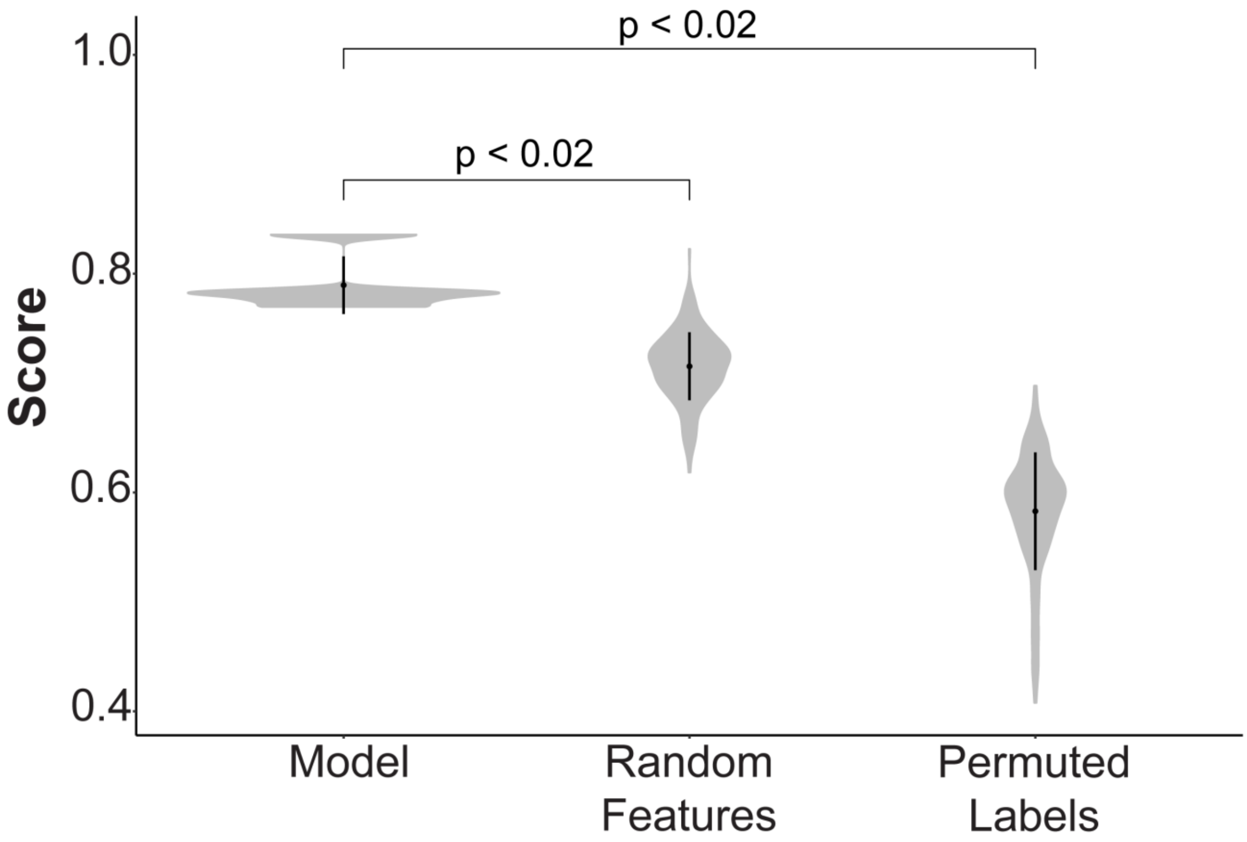

Supplementary Figure 3. Validation of PLS-DA generated model of distinguishing humoral features at < 1 Week and 2-3 Weeks post-infection. The selected features in Figure 4 B-D were compared for validity against random features and permuted labels. A total of 10 random features and 10 permutations were used in the model validation (see methods).
