# Supplementary material for: Anamnestic humoral correlates of immunity across SARS-CoV-2 variants of concern
Source: mBio. 2023 Aug 3;14(4):e00902-23. doi: 10.1128/mbio.00902-23 (PMC10470538; doi:10.1128/mbio.00902-23)
Supplement: Figure S4 — S2-expansion is observed in independent cohorts. [file mbio.00902-23-s0004.pdf]

**Figure S4**

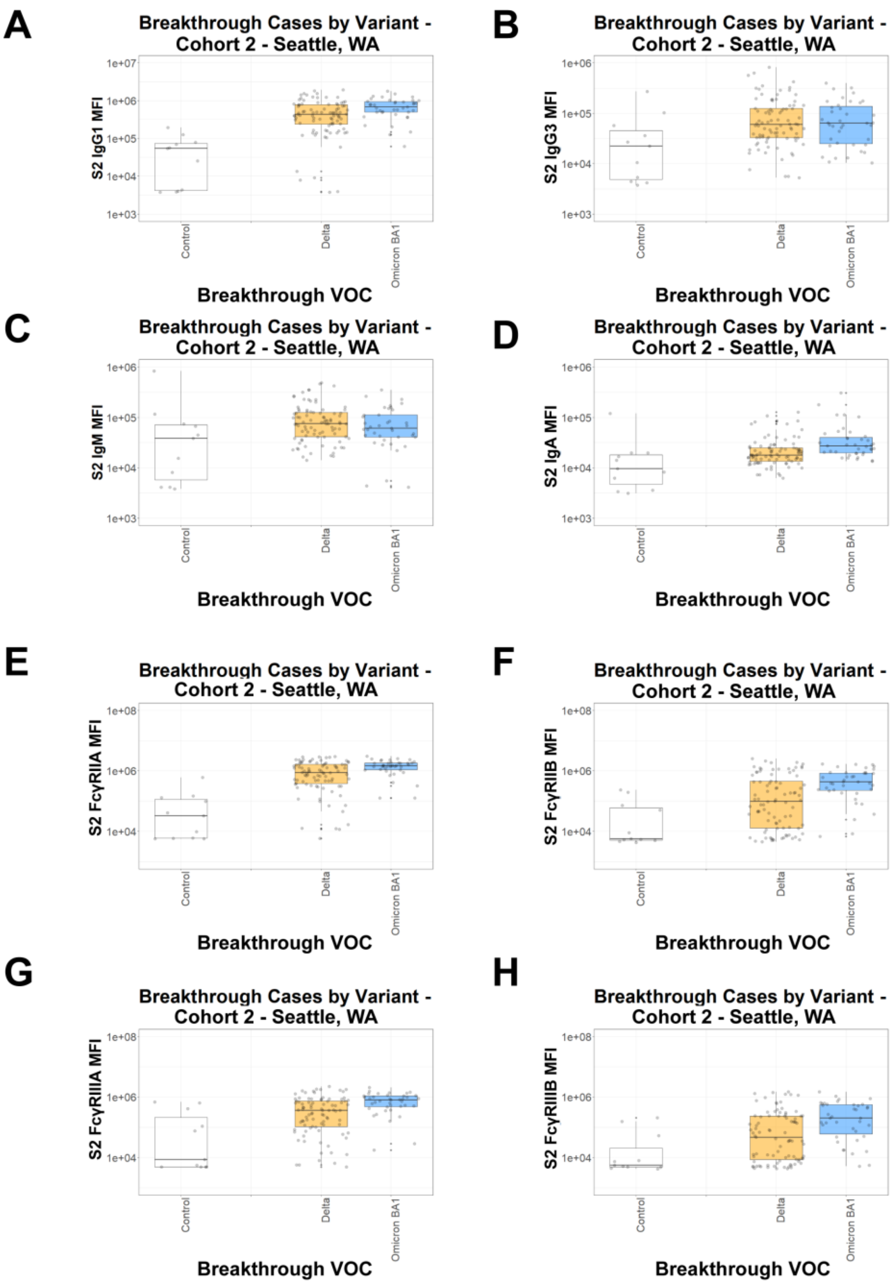


**Supplementary Figure 4. S2-expansion is observed in independent cohorts.** (A) Uninfected controls (column 1, white), Delta breakthrough cases (column 2, orange), and Omicron BA.1 (blue, column 3) were assayed for S2-specific IgG1. Sera were harvested 30 days after diagnosis. (B) Same as A, but for IgG3. (C) Same as A, but for IgM. (D) Same as A, but for IgA. (E) Same as A, for S2-specific FcγRIIA responses. (F) Same as E, but for FcγRIIB. (G) Same as E, but for FcγRIIIA**.** (H) Same as E, but for FcγRIIIB.
