# Supplementary material for: Anamnestic humoral correlates of immunity across SARS-CoV-2 variants of concern
Source: mBio. 2023 Aug 3;14(4):e00902-23. doi: 10.1128/mbio.00902-23 (PMC10470538; doi:10.1128/mbio.00902-23)
Supplement: Figure S5 — Correlations between neutralization and Spike domains are focused on the RBD of D614G and Delta, but not S2, post-breakthrough. [file mbio.00902-23-s0005.pdf]

Figure S5

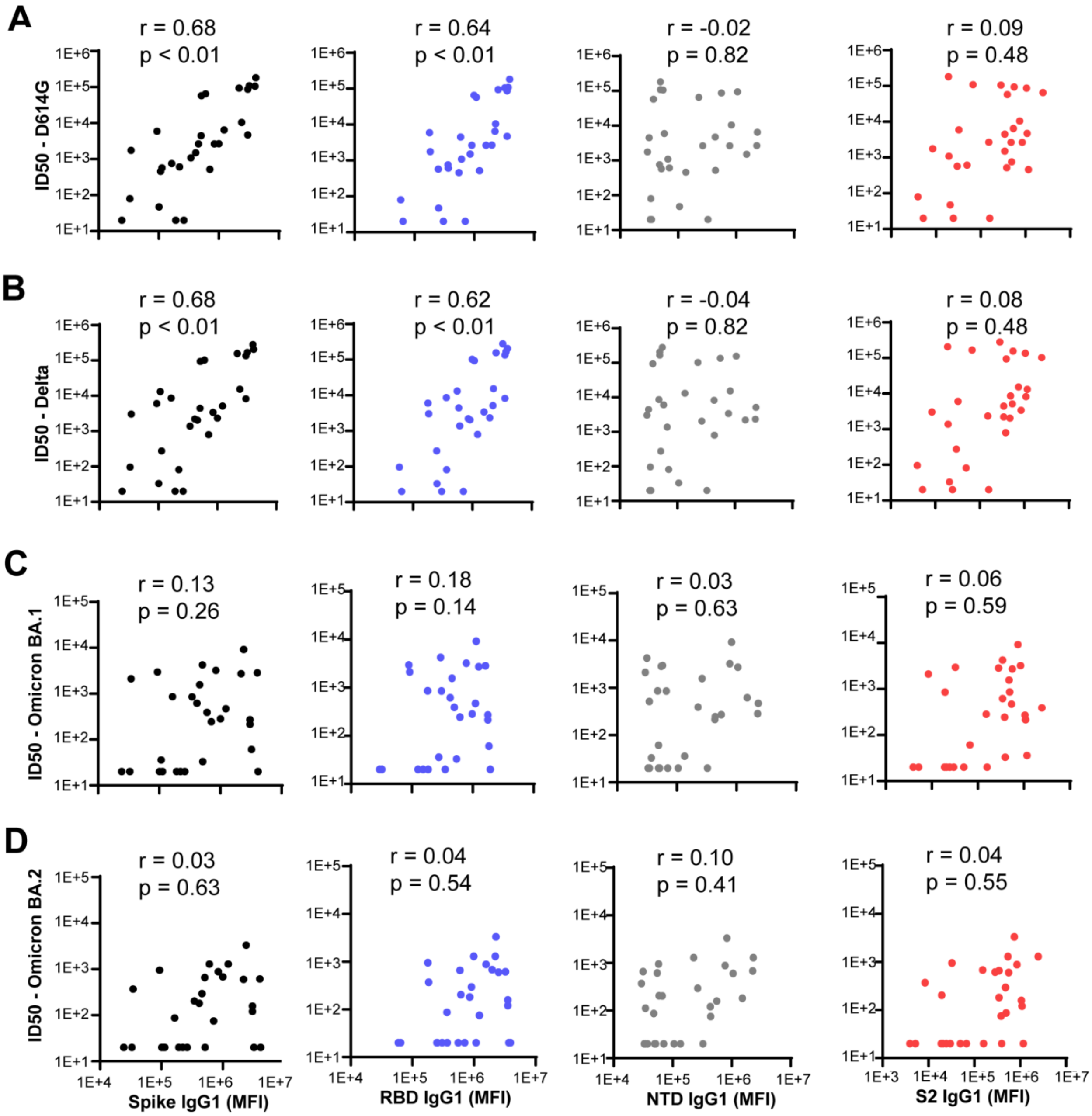

Supplementary Figure 5. Correlations between neutralization and Spike domains are focused on the RBD of D614G and Delta, but not S2, post-breakthrough. (A) Vaccinated individuals with breakthrough COVID-19 were assayed for correlation between neutralization (ID50) and IgG1 Spike (black), RBD (blue), NTD (gray), or S2 (red). Shown above each panel are correlation coefficients and p-values. (B) Same as A, but for neutralization of Delta. (C) Same as A, but for neutralization of Omicron BA.1. (D) Same as A, but for neutralization of Omicron BA.2
