# Supplementary material for: Anamnestic humoral correlates of immunity across SARS-CoV-2 variants of concern
Source: mBio. 2023 Aug 3;14(4):e00902-23. doi: 10.1128/mbio.00902-23 (PMC10470538; doi:10.1128/mbio.00902-23)
Supplement: Figure S6 — Humoral S2 subregion expansions are inversely correlated with viral loads. [file mbio.00902-23-s0006.pdf]

Figure S6

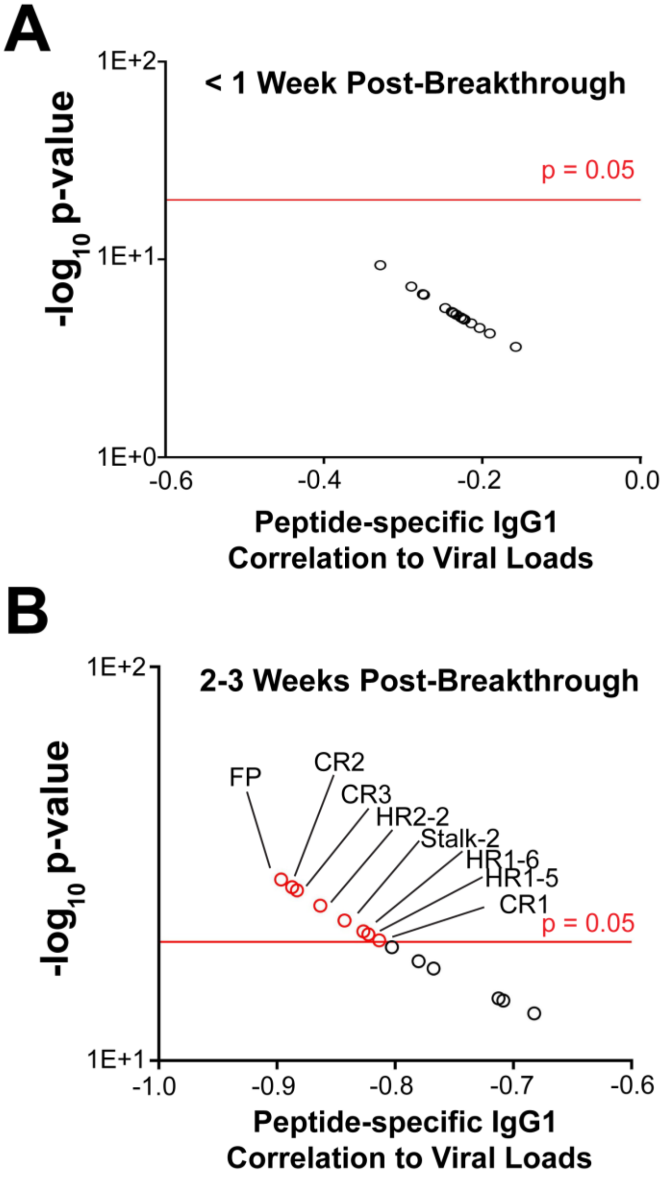

Supplementary Figure 6. Humoral S2 subregion expansions are inversely correlated with viral loads. (A) Correlations between viral loads and IgG1 S2 peptide binding (in MFI) for the < 1 Week post-infection samples. The x-axis represents the Spearman's correlation coefficient between the mean viral loads at < 1 Week and the MFI of IgG1 binding to the peptides. An x-axis value of +1 indicates a tight positive correlation, and a value of -1 indicates a strong inverse correlation. The y-axis is the $-\log_{10}$ p-value of the correlation. Therefore, values higher on the y-axis are lower in value. A solid red line indicating $p = 0.05$ is shown. (B) Same as A, but for the correlations between IgG1 binding to S2 peptides and viral loads at 2-3 Weeks post-infection. All S2 features with a p-value < 0.05 are shaded red and labeled with the corresponding peptide region.
