# Supplementary material for: Anamnestic humoral correlates of immunity across SARS-CoV-2 variants of concern
Source: mBio. 2023 Aug 3;14(4):e00902-23. doi: 10.1128/mbio.00902-23 (PMC10470538; doi:10.1128/mbio.00902-23)
Supplement: Table S1 — Cohort analysis by VOC breakthrough and vaccine type. [file mbio.00902-23-s0007.docx]

**Table S1**

|  | **Control (Vaccinated, non-breakthrough)** | **Vaccinated, Delta VoC Breakthrough** | **Vaccinated, Omicron VoC Breakthrough** |
| --- | --- | --- | --- |
| N | 11 | 37 | 19 |
| Peak Viral Loads  (log10) | N/A/ | 6.2 ± 1.5 | 5.9 ± 1.6 |
| Fully Vaccinated | 11 | 28 | 18 |
| Breakthrough from mRNA-1273 | N/A | 6 | 6 |
| Breakthrough from BNT162b2 | N/A | 14 | 9 |

**Table S1.** Cohort analysis by VOC breakthrough and vaccine type. Other vaccinations included Ad26.S and unknown.
