# Supplementary material for: Anamnestic humoral correlates of immunity across SARS-CoV-2 variants of concern
Source: mBio. 2023 Aug 3;14(4):e00902-23. doi: 10.1128/mbio.00902-23 (PMC10470538; doi:10.1128/mbio.00902-23)
Supplement: Table S2 — Peptide names and sequences used for this study. [file mbio.00902-23-s0008.docx]

**Table S2**

| **Peptide Name** | **Sequence** |
| --- | --- |
| FP | DPSKPSKRSFIEDLLFNKV+SFIEDLLFNKVTLADA |
| CR1 | QYGDCLGDIAARDLICAQKFNG |
| CR2 | LTDEMIAQYTSALLAGTITSGWTFGAGA |
| CR3 | IPFAMQMAYRFNGIG |
| HR1-3 | KLIANQFNSAIGKIQDSLSSTASALGKLQDVVNQN |
| HR1-4 | ALGKLQDVVNQNAQALNTLVKQLSSN |
| HR1-5 | AISSVLNDILSRLDKVEAEVQ |
| HR1-6 | QLSSNFGAISSVLNDILSRLDKVEAEVQIDRLITGRLQS |
| HR1-7 | SVLNDILSR |
| Stalk-1 | DPLQPELDSFKEELDKYFKNHTSPD |
| Stalk-2 | SFKEELDKYFKNHTS |
| HR2-1 | ASVVNIQKEIDRLNEVAKNLNESLIDLQELGKYEQ |
| HR2-2 | QKEIDRLNEVAKNLNESLIDLQE |

**Table S2.** S2 Peptide names and sequences used for this study.
